# Replication and Active Partition of Integrative and Conjugative Elements (ICEs) of the SXT/R391 Family: The Line between ICEs and Conjugative Plasmids Is Getting Thinner

**DOI:** 10.1371/journal.pgen.1005298

**Published:** 2015-06-10

**Authors:** Nicolas Carraro, Dominique Poulin, Vincent Burrus

**Affiliations:** Laboratory of bacterial molecular genetics, Département de biologie, Faculté des sciences, Université de Sherbrooke, Sherbrooke, Québec, Canada; Harvard University, UNITED STATES

## Abstract

Integrative and Conjugative Elements (ICEs) of the SXT/R391 family disseminate multidrug resistance among pathogenic *Gammaproteobacteria* such as *Vibrio cholerae*. SXT/R391 ICEs are mobile genetic elements that reside in the chromosome of their host and eventually self-transfer to other bacteria by conjugation. Conjugative transfer of SXT/R391 ICEs involves a transient extrachromosomal circular plasmid-like form that is thought to be the substrate for single-stranded DNA translocation to the recipient cell through the mating pore. This plasmid-like form is thought to be non-replicative and is consequently expected to be highly unstable. We report here that the ICE R391 of *Providencia rettgeri* is impervious to loss upon cell division. We have investigated the genetic determinants contributing to R391 stability. First, we found that a *hipAB*-like toxin/antitoxin system improves R391 stability as its deletion resulted in a tenfold increase of R391 loss. Because *hipAB* is not a conserved feature of SXT/R391 ICEs, we sought for alternative and conserved stabilization mechanisms. We found that conjugation itself does not stabilize R391 as deletion of *traG*, which abolishes conjugative transfer, did not influence the frequency of loss. However, deletion of either the relaxase-encoding gene *traI* or the origin of transfer (*oriT*) led to a dramatic increase of R391 loss correlated with a copy number decrease of its plasmid-like form. This observation suggests that replication initiated at *oriT* by TraI is essential not only for conjugative transfer but also for stabilization of SXT/R391 ICEs. Finally, we uncovered *srpMRC*, a conserved locus coding for two proteins distantly related to the type II (actin-type ATPase) *parMRC* partitioning system of plasmid R1. R391 and plasmid stabilization assays demonstrate that *srpMRC* is active and contributes to reducing R391 loss. While partitioning systems usually stabilizes low-copy plasmids, *srpMRC* is the first to be reported that stabilizes a family of ICEs.

## Author Summary

Integrative and conjugative elements (ICEs) constitute a class of mobile genetic elements defined by their ability to integrate into the chromosome of their host cell and to transfer by conjugation. Some of the most studied ICEs belong to the SXT/R391 family, which are major drivers of multidrug resistance dissemination among various pathogenic *Gammaproteobacteria*. Transfer of SXT/R391 ICEs to a new host first requires its excision from the chromosome as a circular molecule, which may be lost if the cell divides. *In silico* analyses revealed several putative stabilization systems carried by R391, a prototypical member of the SXT/R391 ICEs family originally isolated from *Providencia rettgeri*. We discovered that, besides stabilization by integration into the chromosome, stability of SXT/R391 ICEs also depends on toxin/antitoxin systems and plasmid-like features including intracellular replication and active partition. Thus, although it has been known for a long time that ICEs and conjugative plasmids use similar strategies to transfer between bacterial populations, our work reveals additional unforeseen similarities in their mechanisms of maintenance in the host cell.

## Introduction

Integrative and conjugative elements (ICEs) are highly prevalent and widely distributed in bacterial genomes [1–3]. Their ability to self-transfer by conjugation between genetically unrelated bacteria contributes to the emergence of multidrug resistant pathogens in diverse taxonomic groups [4–6]. ICEs usually reside within and replicate with the host cell’s chromosome to be vertically inherited. ICEs eventually excise from the chromosome and form circular covalently closed molecules that serve as the substrate for the conjugative machinery that translocates the ICE DNA to recipient cells [6, 7]. With a few exceptions reported only for ICEs of *Actinobacteria*, this conjugative machinery usually consists of a relaxase, a type IV coupling protein and a type IV secretion system [1–3, 8].

The SXT/R391 family of ICEs encompasses one of the largest and most diverse set of ICEs studied, including elements that have been found over the past 40 years in clinical and environmental isolates of diverse species of *Gammaproteobacteria* [9, 10]. ICEs of the SXT/R391 family largely contribute to the spread of antibiotic resistance genes in the seventh-pandemic lineage of *Vibrio cholerae*, the etiologic agent of cholera, which remains a major cause of mortality and morbidity on a global scale [11]. The ICE SXT is a prototypical member of the SXT/R391 family originally isolated from a 1992 Indian multidrug resistant clinical isolate of *V*. *cholerae* O139 [12]. SXT and several variants detected in *V*. *cholerae* O139, O1 and non-O1 non-O139 isolates confer resistance to sulfamethoxazole, trimethoprim, streptomycin and chloramphenicol [9]. The second prototypical member of this family is R391, which was originally detected in a 1967 South African isolate of *Providencia rettgeri* [13]. R391 confers resistance to kanamycin and mercury. Members of the SXT/R391 family all share a common integration site, the 5’ end of *prfC*, and a highly conserved core of genes and sequences that mediate their regulation, integration/excision and conjugative transfer [10]. Expression of the conjugative function of SXT/R391 ICEs is tightly regulated by SetR, which represses the expression of the master activator genes *setC* and *setD*. Their products activate transcription of *int*, *xis* and conjugation-associated operons [14]. Repression of *setC* and *setD* is alleviated by induction of the bacterial response to DNA damage, which promotes autoproteolysis of SetR [15].

SXT and R391 can exist co-integrated in a tandem fashion in *prfC* in the same host cell [16, 17]. Such tandem arrays are suitable substrates for frequent homologous recombination events yielding hybrid ICEs that can be easily segregated in exconjugant cells [16, 18]. Interestingly, R391 was reported to be found as a circular extrachromosomal replicative form in a *recA* recipient strain bearing an integrated copy of R997, another SXT/R391 ICE found in *Proteus mirabilis* [19]. A similar behavior was also reported for R997 entering a *recA* recipient bearing an integrated R391. However, no extrachromosomal form of R391 or R997 could be recovered from *recA* ^+^ hosts. These observations suggest that, at least in specific circumstances, SXT/R391 ICEs are capable of autonomous replication. Autonomous replication was previously suspected for several ICEs and recently well characterized for ICE*Bs1*, an ICE of the Gram-positive bacterium *Bacillus subtilis* [20–25]. Plasmid-like replication was also shown to be essential for the stability of ICE*Bs1* [24]. However, whether autonomous replication is relevant to the biology and stability of SXT/R391 ICEs remains to be established.

Breaking with old paradigms about ICEs, we report here that replication is a key step of the lifecycle of SXT/R391 ICEs by using R391 as a model. By monitoring the frequency of excision, the ICE copy number as well as the frequency of loss of a set of mutants, we show that the putative relaxase TraI and the origin of transfer (*oriT*) are essential for R391 replication and its stability in the progeny of host cells. Furthermore, we demonstrate that, besides diverse non-conserved toxin-antitoxin systems, all SXT/R391 ICEs also encode a conserved plasmid-like type II partitioning system that enhances their stability. Together, these results unravel an unforeseen similarity between the biology of ICEs and conjugative plasmids.

## Results

### Dynamics of R391: Evidence for autonomous replication

**Fig 1:**
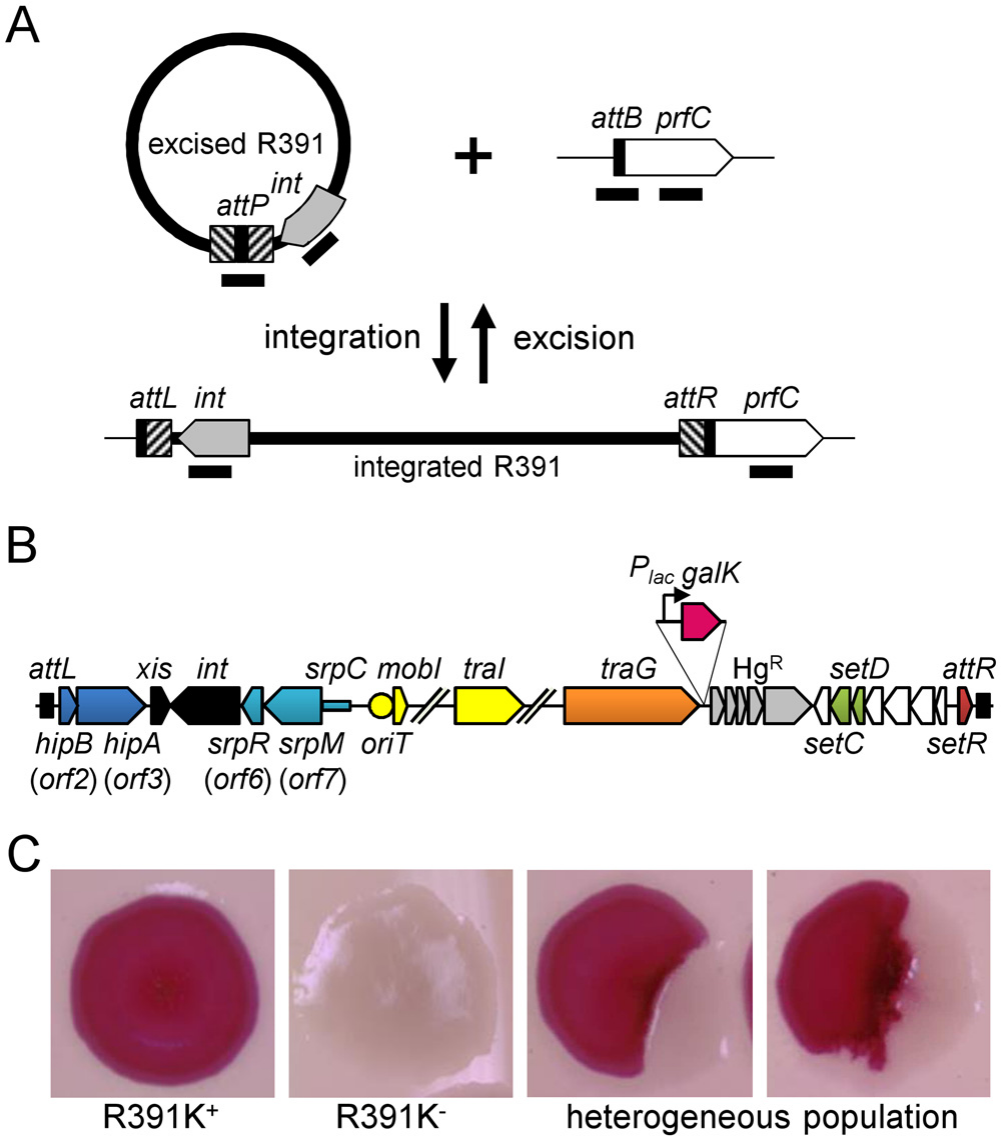
Genetic determinants of R391 stability. (A) Quantification of R391 integration/excision and replication. The DNA targets (*attP*, *attB*, *int* and *prfC*) measured by real-time quantitative PCR are shown by black bars. (B) Schematic representation of R391 and R391K structures. The position and orientation of open reading frames (ORFs) are indicated by arrowed boxes. The colors depict the function deduced from functional analyses and BLAST comparisons: dark blue, toxin/antitoxin system; black, site-specific recombination; light blue, active partitioning system; yellow, DNA processing; orange; mating pair formation; light grey, mercury resistance (Hg^R^); green, transcriptional activators; red, transcriptional repressor; white, other or unknown functions. The putative centromere-like region (*srpC*) and the origin of transfer (*oriT*) are depicted by a light blue rectangle and a yellow circle, respectively. *hipAB* and the mercury resistance genes are not conserved features of SXT/R391 ICEs. To construct R391K, the reporter gene *galK* (in magenta) under the control of the IPTG-inducible *P*_*lac*_ promoter was inserted between *traG* and *merR*, the first gene of the mercury resistance operon. (C) Colony color-based assay for the detection of R391K-containing colonies on MacConkey D-galactose indicator agar. Colonies bearing *galK*-tagged R391 (R391K) appeared red on the indicator medium (R391K^+^) while white colonies lacked the ICE (R391K^-^). Sectored colonies forming during growth on agar plates are indicative of high instability of R391K mutants.

**Fig 2:**
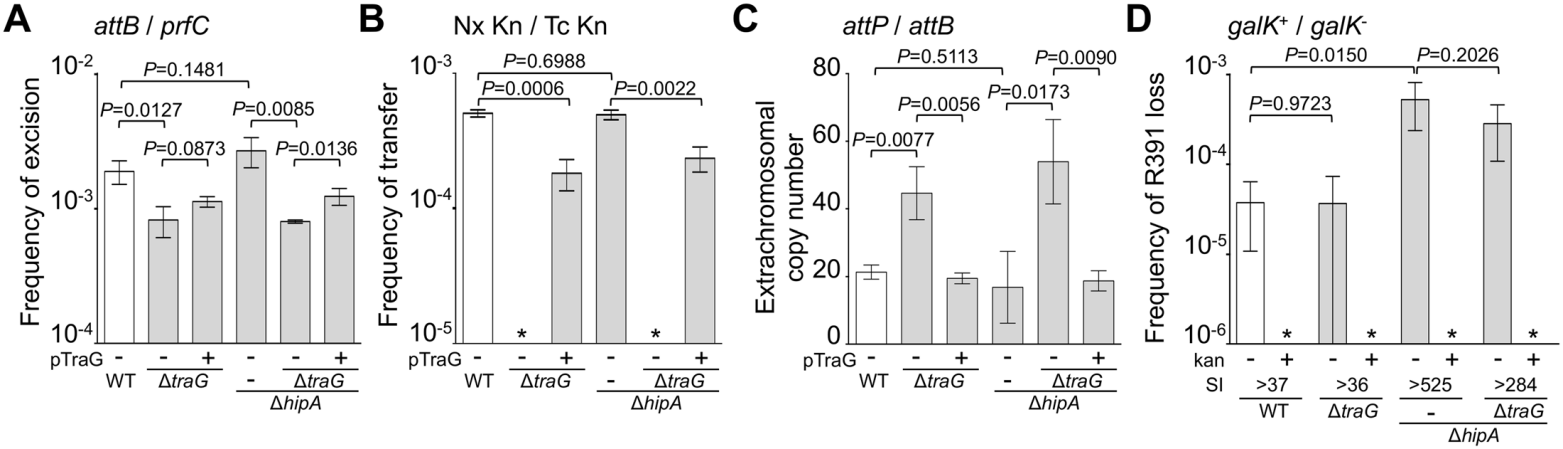
Effects of the deletion of *hipA* or abolition of conjugative transfer (Δ*traG*) on R391 dynamics. (A) Effect on R391K excision. The frequency of excision of the wild-type R391K ICE (WT) or its Δ*hipA* and Δ*traG* mutants corresponds to the *attB*/*prfC* ratio measured by qPCR. (B) Effect on R391K transfer. Conjugation assays were carried out using *E*. *coli* VB38 containing R391K (WT) or its Δ*hipA* and Δ*traG* mutants as donors. *E*. *coli* MG1655 Nx (VB111) was used as the recipient. Transfer frequencies are expressed as the number of exconjugant per donor CFUs. The asterisk indicates that the frequency of exconjugant formation was below the detection limit (<10^-8^). (C) Effect on the copy number of excised R391K. Extrachromosomal copy numbers of the wild-type R391 (WT) and its Δ*hipA* and Δ*traG* mutants correspond to *attP*/*attB* ratios measured by qPCR. (D) Effect on R391K stability. The frequency of loss of wild-type R391K (WT) or its Δ*hipA* and Δ*traG* mutants was measured as the ratio between the number of R391K^-^ (white colonies) and the number of R391K^+^ (red colonies) CFUs on MacConkey galactose agar plates after 16 hours of growth in LB medium with (+) or without (-) antibiotic. The stability index (SI) corresponds to the ratio of the frequencies of loss observed with and without antibiotic. The asterisk indicates that the frequency of loss was below the detection limit (<10^-6^). For panels A, B and C, complementation of the Δ*traG* mutation was carried out using *traG* expressed from an arabinose-inducible promoter (*P*_*BAD*_) provided by pTraG. In all panels, the bars represent the means and standard deviation values obtained from at least three independent experiments. Statistical analyses were carried out using two-tailed Student’s *t*-tests and the *P*-value is indicated above the brackets comparing two bars.

To have a better understanding of SXT/R391 ICEs biology, we evaluated five key factors of R391 lifecycle in *Escherichia coli*: (i) the dynamics of excision/integration, which will be reported as the frequency of excision in the rest of the manuscript, (ii) the frequency of transfer, (iii) the average copy number per cell in the whole cell population, (iv) the average number of extrachromosomal circular copies per cell, and (v) the ICE stability in the cell population. The frequency of R391 excision was assessed by quantifying by real-time quantitative PCR (qPCR) the relative amount of free integration site (*attB*) resulting from R391 excision per chromosome as measured by the amount of chromosomal *prfC* target (Fig 1A). R391 excised at a frequency of 1.90×10^-3^ ± 0.38×10^-3^ (Fig 2A), which is about tenfold lower than the excision frequency of SXT (1.76×10^-2^ ± 0.65×10^-2^, *P* = 0.0140, two-tailed Student *t*-test) in similar conditions. Mating assays showed that R391 transfers at about 5.01×10^-4^ ± 0.31×10^-4^ exconjugant/donor (Fig 2B), which is about 20 fold higher than SXT (2.71×10^-5^ ± 0.55×10^-5^ exconjugant/donor). Hence there is no correlation between the frequency of excision of these elements measured in the whole cell population and their respective frequency of transfer. This observation indicates that excision is not a factor that limits the rate of dissemination of these two ICEs.

Using the same approach, we then measured the mean copy number per cell of R391 in the whole cell population as the ratio between the amount of R391-borne *int* target and the amount of chromosomal *prfC* target (Fig 1A). This ratio was found to be 0.96 ± 0.04 as expect for a single copy of R391 integrated in the chromosome. We also measured the mean copy number of the extrachromosomal circular form of R391 per cell by establishing the ratio between the amount of *attP* recombination site resulting from R391 excision and the amount of unoccupied chromosomal *attB* sites. In theory, each event of R391 excision is expected to yield one unoccupied *attB* site on the chromosome and one *attP* site on the circular excised R391 (*attP*/*attB* = 1). We observed that this ratio reached 21 ± 2 (Fig 2C), suggesting that R391 is capable of replicating in a small subset of the cell population in which it is excised from the chromosome. This observation is consistent with results previously reported for SXT, for which 4 *attP* sites on average exist for each unoccupied *attB* site [16, 26]. New measurements carried out in this study to confirm these reports revealed 3.6 ± 0.2 *attP* sites per unoccupied *attB* site for SXT.

We then assessed the stability of R391 by monitoring the number of cells lacking R391K in the cell population after 16 hours of growth (about 20 generations) in LB medium with or without selective pressure. R391K is tagged with the *galK* reporter gene under the control of the *P*_*lac*_ promoter to enable high-level galactokinase activity in a *lacI* mutant strain such as *E*. *coli* VB38 [18], a Δ*galK* derivative of *E*. *coli* CAG18439 (*lacI42*::Tn*10*) (Fig 1B). The frequency of loss was determined as the percentage of white colonies (*galK* ^-^, devoid of R391K) on MacConkey indicator agar supplemented with 1% galactose (Fig 1C). R391K was found to be inherently stable because it was lost in only 0.0037% of the cell population in the absence of selective pressure (Fig 2D, WT), whereas no detectable loss was observed when cells were grown with kanamycin in liquid culture (detection limit of 0.0001%).

### A HipAB-like toxin-antitoxin system stabilizes R391

Stability of many mobile genetic elements relies on a post-segregational killing mechanism, which induces a strong selective disadvantage or even death to cells that have lost them [27–31]. While previous studies have shown that two functional toxin-antitoxin (TA) systems, *mosAT* and *s045*-*s044*, enhance the stability of SXT [32, 33], neither of these TA systems was found in R391. Nevertheless, *in silico* analysis of the R391 sequence revealed that the two overlapping open reading frames (ORFs) *orf02* and *orf03*, which belong to the variable region I located upstream of *xis*, encode a putative *hipAB*-like TA system (Fig 1B) [34, 35]. Indeed, *orf03* (*hipA*) is predicted to encode a HipA-like toxin, while *orf02* (*hipB*) likely codes for the HipB cognate antitoxin, which carries a DNA-binding HTH-XRE (HTH_19) domain. To measure the impact of this putative *hipAB*-like TA system on R391 stability, we constructed a Δ*hipA* mutant of R391K. This mutation did not impair the transfer of R391K and had no effect on the excision or extrachromosomal copy number of the element (Fig 2A, 2B and 2C, compare WT and Δ*hipA*). However, ICE stability was affected as R391K loss increased by 12 fold for the Δ*hipA* mutant compared to wild-type (Fig 2D). No loss of R391K Δ*hipA* was detectable in the presence of kanamycin. These results revealed the functionality of the *hipAB* TA system and its involvement in the stability of R391, as previously demonstrated for *mosAT* of SXT [32]. However, like the *mosAT* and *s045*-*s044* loci of SXT, *hipAB* of R391 is not a conserved feature of SXT/R391 ICEs; therefore *hipAB* is likely not an inherent and ancestral mechanism used by SXT/R391 ICEs to enhance their stability in their respective hosts. Cell death or growth reduction associated with *hipAB* after R391 loss was likely to hinder our investigations on R391 stability. To circumvent this issue, the Δ*hipA* mutant provided us with a useful tool for additional investigations aimed at unraveling other stabilization mechanisms conserved among ICEs of the SXT/R391 family.

### Conjugation does not stabilize SXT/R391 ICEs

Conjugation has been shown to be a powerful stabilization mechanism for conjugative plasmids that can reenter in cells having lost them by infectiously spreading in the cell population [28, 36]. While Wozniak and Waldor [32] have shown that conjugation does not promote SXT loss, their assay does not allow to conclude whether conjugation is an efficient mechanism of stabilization of SXT/R391 ICEs. To answer this question, we looked at the frequency of loss of a Δ*traG* mutant of R391K. *traG* codes for an inner-membrane component of the donor cell mating pair formation apparatus that is essential for SXT transfer [37]. As previously reported for SXT, deletion of *traG* abolished R391K transfer (Fig 2B). However, the inability to transfer did not reduce the stability of R391K, as the frequencies of loss of wild-type R391K and its Δ*traG* mutant were nearly identical (Fig 2D). No detectable loss of R391K Δ*traG* was observed when kanamycin was added during liquid culture. The frequencies of excision of the Δ*traG* and Δ*hipA* Δ*traG* mutants were reduced by ~2.3 fold, while their extrachromosomal copy numbers were more than twice as high as wild-type R391K and its Δ*hipA* mutant, reaching up to ~54 copies per cells (Fig 2A and 2C). This observation suggests that, once excised from the chromosome, the circular form of R391K accumulates in the cell possibly because a defective mating apparatus caused by the Δ*traG* mutation cannot mediate its transfer to a recipient cell.

### The putative relaxase TraI is key for transfer, replication and stability of R391

The plasmid-like replication of ICE*Bs1* was shown to be essential for its stability [24]. Rolling-circle replication of ICE*Bs1* requires the relaxase NicK and *oriT*, a *cis*-acting locus initiating the translocation of DNA through the mating pore.

**Fig 3:**
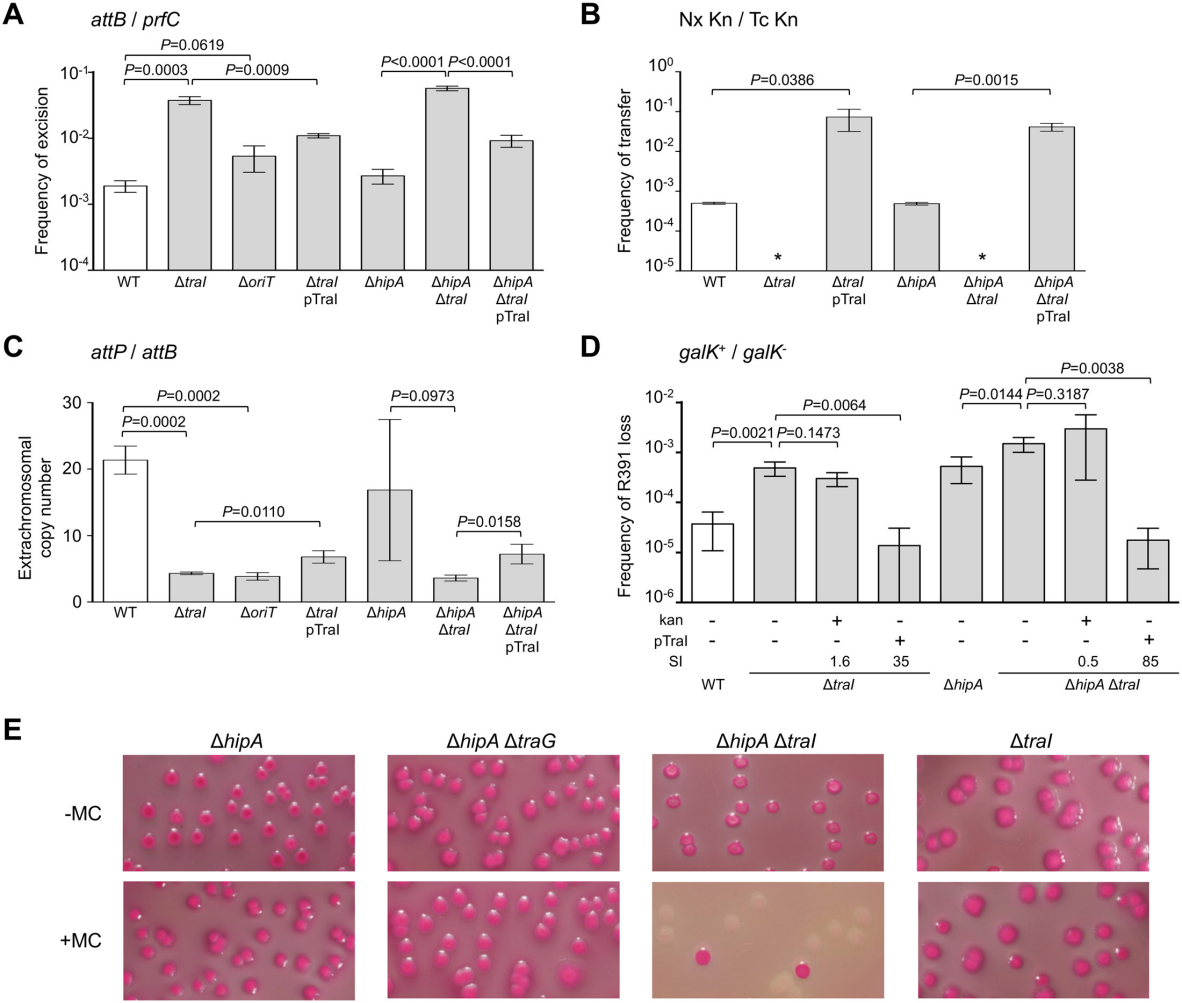
*traI* and *oriT* are key factors for R391 replication and stability. (A) Effect of *traI* or *oriT* deletions on R391K excision. (B) Effect of *traI* deletion on R391K transfer. (C) Effect of *traI* and *oriT* deletions on the copy number of excised R391K. (D) Effect of *traI* deletion on the stability of R391K. For all panels, the experiments were carried out as described in Fig 2. Complementation of the Δ*traI* mutation was carried out using *traI* expressed from an arabinose-inducible promoter (*P*_*BAD*_) provided by pTraI. (E) Effect of mitomycin C on the stability of R391K mutants observed on MacConkey galactose agar after 16-hour growth in LB medium with (+) or without (-) mitomycin C (MC).

In a subset of a cell population, R391 seems to be in a multicopy plasmid-like state that may be important for preserving ICE stability when it is excised from the chromosome in actively dividing cells. To test this hypothesis, *oriT* and *traI* deletion mutants were constructed in R391K (Fig 1B). *traI* codes for the putative relaxase of SXT/R391 ICEs that recognize the origin of transfer (*oriT*) [38]. As expected, the Δ*traI* mutation abolished R391K conjugative transfer (Fig 3B). We also observed a ~5-fold reduction of the extrachromosomal copy number of R391K when either *traI* or *oriT* were missing compared to wild-type (Fig 3C). The Δ*traI* mutation also led to a ~20-fold increase of the frequency of excision and to an 11-fold increase of R391K loss (Fig 3A, 3B and 3D). Combined Δ*traI* and Δ*hipA* mutations led to a 34-fold increase of R391K loss, thereby confirming that *traI* is important for R391 stability (Fig 3D). Expression of *traI* in *trans* from the arabinose-inducible *P*_BAD_ promoter in pTraI restored and even enhanced the transfer and the stability of both Δ*traI* and Δ*traI* Δ*hipA* mutants compared to wild-type (Fig 3B and 3D). Although the copy number of the plasmid-like form of R391K Δ*traI* doubled upon complementation with pTraI, it failed to reach the wild-type level (Fig 3C). Interestingly, Δ*traI* mutants were so unstable that selective pressure exerted by kanamycin in liquid culture did not, or only slightly, improved R391K stability (Fig 3D). The high instability affecting Δ*traI* mutants also led to the formation of sectored colonies on agar plates likely resulting from loss of R391K during colony development (Fig 1C).

Since conjugative transfer of SXT/R391 ICEs is known to be stimulated by DNA-damaging agents, we tested the effect of mitomycin C on the stability of the Δ*hipA*, Δ*traG* and Δ*traI* mutants of R391K. We observed that the drug did not induce high-frequency loss of the Δ*hipA*, Δ*hipA* Δ*traG* or Δ*traI* mutants of R391K (Fig 3E). In striking contrast, deletion of both Δ*hipA* and Δ*traI* led to a hypersensitivity of R391K to mitomycin C treatment as the ICE was lost in more than 90% of the cell population (Fig 3E). We suspect that Δ*traI* mutants are highly unstable; yet in the presence of *hipAB*, cells that have lost R391K Δ*traI* likely have no progeny or strong growth reduction due to the persistence of the HipA toxin, thereby masking this high instability in conditions that strongly induce R391 excision.

### SXT/R391 ICEs encode a functional plasmid-like active partition system

*In silico* analysis of R391 sequence using CD-search on the Conserved Domain Database v3.11 [39, 40] and protein fold recognition server Phyre2 [41] revealed that *orf07*, the first gene of an operon containing *int*, codes for a predicted actin-like NTPase structurally related to the ParM plasmid segregation proteins of plasmids R1 and pSK41 (Fig 1B). ParM proteins are a key component of type II ParMRC partitioning systems that mediate plasmid DNA segregation during cell division via a pushing mechanism [42, 43]. ParR adaptor protein connects *parC*, a *cis*-acting centromere-like locus, to the ParM filament. ParR proteins have low conservation and their genes are found downstream of the *parM* gene. The open reading frame *orf06*, located downstream of *orf07*, is predicted to code for a small basic protein (pI 9.3) with no recognizable domain (Fig 1B). Hence, *orf06* may encode a ParR DNA-binding protein that binds the centromere-like region in partitioning systems. Based on these observations and results described below, *orf06* and *orf07* were renamed *srpR* and *srpM* for SXT/R391 ICEs partitioning proteins R and M, respectively (Fig 1B). By functional analogy with the *parMRC* partitioning systems carried by the plasmid R1 of *E*. *coli* and the staphylococcal plasmid pSK41 [42], the DNA fragment located upstream of *srpM* likely corresponds to the centromere-like region bound by SrpR and was annotated *srpC* (Fig 1B). The *srpMRC* locus is strictly conserved in all SXT/R391 ICEs, suggesting that it may somehow play an important role in their biology.

**Fig 4:**
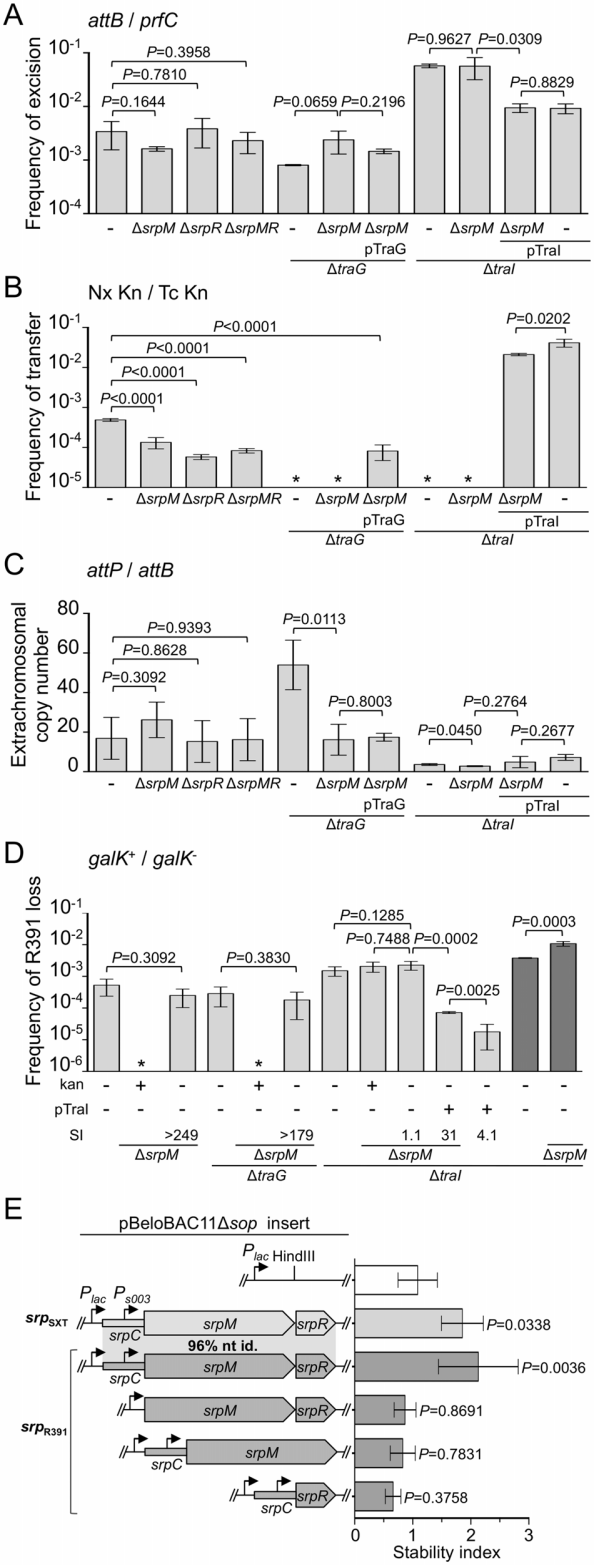
Effect of the SrpMRC active partition system on R391 dynamics. (A) Effect of *srpMRC* inactivation on R391K excision. (B) Effect of *srpMRC* inactivation on R391K transfer. (C) Effect of *srpMRC* inactivation on the copy number of excised R391K. (D) Effect of *srpMRC* inactivation on R391K stability. For panels A to D, experiments were carried out as described in Fig 2 using R391K Δ*hipA* derivatives and complementation of the Δ*traI and* Δ*traG* mutations were carried out using *traI* and *traG* expressed from an arabinose-inducible promoter (*P*_*BAD*_) provided by pTraI and pTraG, respectively. For panel D, stability assays carried out over ~40 generations are indicated by dark grey bars. (E) The *srpMRC* locus stabilizes a partition-deficient single-copy plasmid. The relative stability of the single-copy plasmid pBeloBAC11Δ*sop* and derivatives carrying all or parts of the *srpMRC* locus under control of the *P*_*lac*_ promoter were tested in *E*. *coli* TOP10F’. Expression from *P*_*lac*_ was induced using 0.02 mM of IPTG and cells were grown for 16 hours in M9 minimal medium. The stability index (SI) corresponds to the ratio of the frequencies of loss observed with and without antibiotic (D) or with and without IPTG (E). The bars represent the mean and standard deviation values obtained from at least three independent experiments. Statistical analyses in panel E were performed using one-way ANOVA with Tukey’s multiple comparison test. *P*-values indicated above the bars refer to comparison with pBeloBAC11Δ*sop*.

Deletion of *srpR*, *srpM* or both had no measurable effect on the frequency of excision or the extrachromosomal copy number of R391K Δ*hipA*, and had a slight inhibitory effect (about 4- to 14-fold reduction) on the frequency of transfer (Fig 4A, 4B and 4C). The lack of impact on the excision frequency confirmed that neither deletion has a polar effect on the expression of the *int* gene located immediately downstream of *srpR* (Figs 1B and 4A). Since the deletion of *srpM* had very little effect on R391 transfer, we used this mutation to further study the phenotype associated with a non-functional *srpMRC* locus. Cumulative mutations of *traG*, *hipA* and *srpM* did not affect the frequencies of excision or loss of R391K compared to the Δ*traG* Δ*hipA* mutant (Fig 4A and 4D). However, the extrachromosomal copy number of R391K dropped nearly 4 fold when comparing the same mutants (Fig 4C). Furthermore, a Δ*traI* Δ*hipA* Δ*srpM* triple mutant exhibited a visible but statistically non-significant 35% reduction of stability compared to the Δ*traI* Δ*hipA* mutant (Fig 4D). However, deletion of *srpM* led to a 3-fold increase of the stability of R391K Δ*traI* Δ*hipA* after 40 generations (Fig 4D, dark grey bars). Finally, overexpression of *traI* from pTraI did not completely prevent the loss of R391 Δ*traI* Δ*hipA* Δ*srpM*, which was lost about 4 times more frequently than the Δ*traI* Δ*hipA* mutant (Fig 4D). These data revealed that *srpM* is important for R391 stability when the number of copy of the ICE is low and thus could be a functional active partition system.

To test further whether *srpMRC* is a functional DNA partitioning system, plasmid stabilization assays were carried out using pBeloBAC11Δ*sop*, an unstable derivative of the single-copy plasmid pBeloBAC11 that lacks its native *sopABC* partitioning system. In SXT/R391 ICEs, expression of *srpM*, *srpR* and *int* was shown to be driven from *P*_*s003*_, a promoter exclusively dependent upon activation by the transcriptional activator SetCD (Fig 1B) [14]. To bypass the need for SetCD, the *srpMRC* loci of R391 and SXT were cloned into pBeloBAC11Δ*sop* downstream of the IPTG-inducible *P*_*lac*_ promoter (Fig 4E). Expression of *srpMRC* loci of SXT (pSrp_SXT_) or R391 (pSrp_R391_) upon IPTG induction led to a respective ~1.8 and ~2.1-fold increase of pBeloBAC11Δ*sop* stability, thereby confirming that *srpMRC* is a functional plasmid stabilization system (Fig 4E and S1 Fig). The absence of *srpR*, *srpM* or *srpC* prevented plasmid stabilization, which was then comparable to the empty vector (Fig 4E and S1 Fig).

### SrpR binds the centromere-like sequence *srpC* of SXT/R391 ICEs

**Fig 5:**
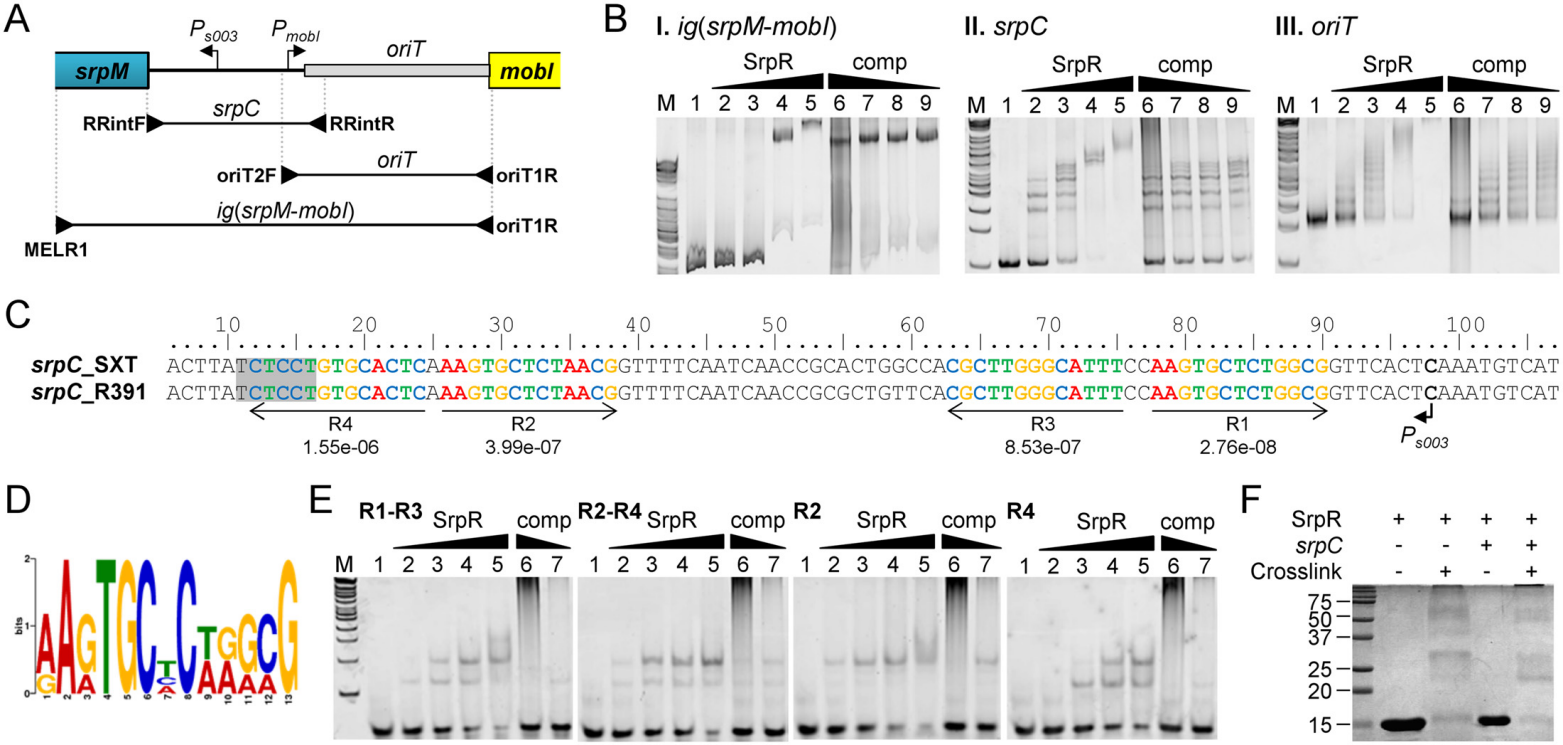
SrpR binds the centromere-like region *srpC*. (A) Schematic representation of the intergenic region between the *srpMR*-*int* operon and *mobI*. The positions of *oriT* and SetCD-dependent promoters *P*_*s003*_ and *P*_*mobI*_ were determined previously [14, 38]. The position of primers used to amplify the probes used in panel B are indicated by arrowheads. (B) Electrophoretic mobility shift assays (EMSA) of DNA fragments of the intergenic region bound by SrpR. Lane M, 2-log DNA ladder; lane 1, 40 ng of DNA probes alone; lanes 2–5, 10, 20, 40, 80 ng of SrpR for *ig*(*srpM*-*mobI*) or 25, 50, 75, 100 ng of SrpR for *oriT* and *srpC*; lanes 6–9, 40 ng (*ig*(*srpM*-*mobI*)), 75 ng (*oriT*) or 50 ng (*srpC*) of SrpR and 200, 40, 20, 10 pg of sonicated salmon sperm DNA as non-specific competitor DNA (comp). (C) Alignment of the complementary strand of the 5’ UTR upstream of *srpM*. The repeated sequences identified by MEME are shown with their respective *p*-values. The shaded area depicts the predicted Shine-Dalgarno sequence of *srpM*. (D) Logo sequence generated from the four repeated sequences shown in C. (E) EMSA of SrpR on the binding sites R1-R3, R2-R4, R2 and R4. Conditions are the same as those used for *srpC* with either 200 or 40 pg of sonicated salmon sperm DNA. (F) SrpR multimerization assays. The size in kDa of the Precision Plus Protein Kaleidoscope Standards (BioRad) is indicated on the left side.

SrpR lacks homologies with known ParR proteins that have been shown to bind *parC*-centromere-like sequences upstream of *parMR* genes in plasmids such as R1. To test whether SrpR is capable of binding the *srpC* locus, we carried out electrophoretic mobility shift essays (EMSA) experiments using purified C-terminally 6xHis-tagged SrpR protein (predicted molecular weight of 10.4 kDa). EMSA assays revealed a specific binding of SrpR to the 615-bp fragment *ig*(*srpM*-*mobI*) which corresponds to the intergenic region between *srpM* and *mobI* and likely contains the *srpC* region (Fig 5A and 5BI). Addition of high concentrations of sonicated salmon sperm DNA (non-specific competitor) did not destabilize SrpR binding to this probe. Further investigation confirmed that SrpR specifically binds the 251-bp *srpC* region as SrpR binding to *srpC* was resilient to the addition of the non-specific competitor DNA (Fig 5A and 5BII). The presence of multiple specific shifts suggests that SrpR binds multiple sites or binds as different multimeric forms (Fig 5BII). While SrpR was able to bind to the 298-bp fragment containing *oriT*, addition of the non-specific competitor DNA destabilized SrpR binding (Fig 5BIII). Non-specific SrpR binding to *oriT* indicates that SrpR exhibits a significant non-specific affinity for DNA molecules.

*In silico* analysis of the centromere-like *srpC* region of R391 and SXT using the Multiple Em for Motif Elicitation tool (MEME) [44] revealed four conserved 13-bp direct and inverted repeats that might be recognized by SrpR (Fig 5C and 5D). EMSA results showed that a 40-bp fragment containing either R1-R3 or R2-R4 was bound by SrpR (Fig 5E). Addition of competitor DNA strongly decreased SrpR binding but did not completely alleviate the interaction. Binding of SrpR to the sequences R2 or R4 used as probes was abolished by the addition of the competitor suggesting that half-sites do not produce stable complexes with SrpR (Fig 5E).

Since ParR-like DNA binding proteins have been shown to form multimeric complexes [45], SrpR multimerization assays were carried out using glutaralehyde cross-linking. These assays suggests that, even without any DNA substrate, SrpR seems to be able to assemble as dimeric and tetrameric complexes in solution as shown by the apparition of large bands migrating at compatible molecular weights in a SDS page gel (Fig 5F). Copious amounts of SrpR were also trapped in the well, thereby suggesting that SrpR could be able to assemble in complexes of higher order.

### SrpMRC of SXT/R391 ICEs is closely related to a putative partitioning system conserved in IncA/C conjugative plasmids

**Fig 6:**
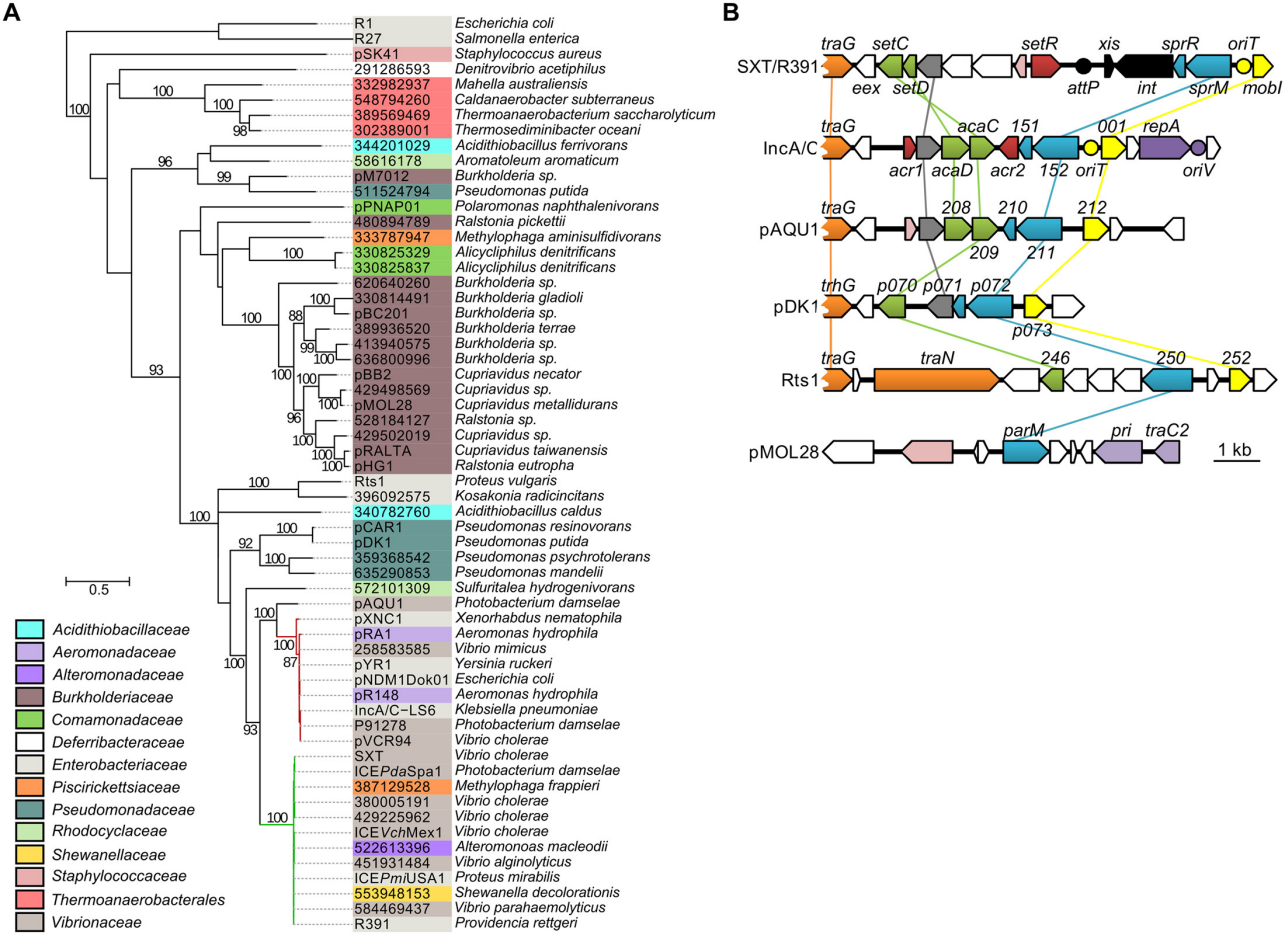
Genetic context and molecular phylogenetic analysis of SrpM. (A) The evolutionary history of SrpM was inferred by using the Maximum Likelihood method based on the Le and Gascuel model [55]. The tree with the highest log likelihood (-15514.1930) is shown. The percentage of trees in which the associated taxa clustered together is shown next to the branches (≥80% cut-off). The background color of each leaf indicates the taxomonic family of original host species from which each element was isolated. SrpM orthologs indicated by their protein sequence GI number are those for which the original element could not be easily identified from data gathered from Genbank and/or for which plasmid naming was inconsistent or not following the rules of Novick et al. [56]. The SXT/R391 ICEs and IncA/C plasmids lineages are shown by green and red branches, respectively. (B) Comparison of the genetic context of genes coding for SrpM orthologs in SXT/R391 ICEs (SXT, AY055428.1), IncA/C plasmids (pVCR94, KF551948), pAQU1 (NC_016983.1), pDK1 (NC_014124.1), Rts1 (NC_003905.1), and pMOL28 (CP000355.2). Arrows of similar color represent genes predicted to have similar functions. Green, transcriptional activator; yellow, MobI-like homologs; red and pink, transcriptional repressor; orange, conjugative transfer; grey, lytic transglycosylase; mauve, replication; black, site-specific recombination; blue, partitioning system; white, other or unknown functions. Yellow circles indicate the position of origins of transfer (*oriT*). The mauve circle indicates the position of the origin of replication (*oriV*) of pVCR94. The black circle indicates the position of the *attP* site for chromosomal integration of SXT by site-specific recombination.

To assess the relationship of SrpMRC with other type II partitioning systems, we carried out a phylogenetic analysis based on the ParM actin-like homologs found by BlastP. Since the ParR adaptor proteins and *parC* sequence usually retain low conservation, they were not included in the analysis. Our analyses revealed that as expected, SrpM of R391 clusters with close orthologs encoded by all SXT/R391 (Fig 6A, green branch). SrpM is also closely related to ParM orthologs encoded by a putative type II partitioning system carried by conjugative plasmids of the IncA/C (Fig 6A, red branch) and pAQU groups [46–48]. SrpM and all of these orthologs cluster with more distantly related plasmids such as Rts1 (IncT) [49] and the catabolic plasmids pCAR1/pDK1 (IncP7) [50, 51]. Interestingly, with the exception of Rts1, which seems to lack a gene coding for a ParR protein, the genetic contexts of the orthologous *parMRC* loci in all these mobile elements are strikingly similar, located between *traG*- and *mobI*-like genes, thereby supporting their common ancestry and divergent evolutionary pathways (Fig 6B). This large group of related *par* loci is distantly related those carried by diverse plasmids broadly distributed among bacterial species of *Delta-* and *Gammaproteobacteria*, including the most distantly related *parMRC* systems carried by the conjugative plasmids R1 and R27 (Fig 6A) [52–54].

## Discussion

In our modern world, antibiotics are widespread in most environments, subjecting microorganisms to a strong and constant selective pressure [57, 58]. ICEs circulating among environmental and pathogenic bacteria can take advantage of this selective pressure by collecting and accumulating antibiotic resistance-conferring genes. The selective advantage conferred by antibiotic resistance enhances the stability of ICEs in their hosts as well as their odds to eventually spread into and invade a new bacterial population. However, ICEs likely predate the antibiotic era and have evolved other means to prevent their loss. Indeed, several ICEs are stably maintained despite the lack of genes coding for any obvious selective advantage for their host [59, 60]. One strategy of stabilization consists in a tight control of the excision of the ICE from the chromosome. However, too tight a regulation could prevent its efficient dissemination. For ICEs of the SXT/R391 family, excision and transfer were shown to be coupled with the activation of the host’s SOS response [15]. In bacteria such as *E*. *coli*, spontaneous induction of the SOS response in the absence of DNA damaging agents has been shown to occur in 0.3 to 3% of the cell population [61], thereby inherently leading to unscheduled excision that is detrimental to ICE stability. Indeed, cell division occurring after ICE excision can generate ICE-free cell lineages, which likely have a competitive advantage in the absence of selective pressure.

Between *attL* and *xis*, R391 bears genes coding for a HipAB-like TA system that enhances the stability to the ICE as inactivation of *hipA* increased R391K loss by 12-fold (Fig 2D). *hipAB* is also found at the same position in ICE*Vch*Mex1, another member of the SXT/R391 family, which does not seem to confer any heavy metal or antibiotic resistance to its original host [10, 60]. The toxin/antitoxin system *mosAT* has been shown to strongly improve the stability of SXT [32]. Interestingly, *mosAT* expression was found to be correlated with activation of SXT excision and conjugative transfer [32]. However, coupling of *mosAT* expression with SXT excision was later shown to be circumstantial to the activation by SetCD of the expression of the upstream *traVA* genes [14]. Furthermore, since neither *mosAT* nor *hipAB* are conserved in all SXT/R391 ICEs [10], element-specific TA systems located in variable regions should only be considered as auxiliary determinants of stabilization for this family of ICEs. The same holds true for the *tad*-*ata*-type TA system *s044*-*s045* carried by SXT in the variable region located between *traIDJ* and *traLEKB* [10, 33].

In addition to diverse TA systems encoded by variable DNA, we have shown here that SXT/R391 ICEs rely on specific and conserved strategies to enhance their stability within their host genome. Besides the most obvious one, which is their integration within the host chromosome, our data support the notion that SXT/R391 ICEs are not only capable of replication, but they can also actively segregate the resulting plasmid-like forms.

**Fig 7:**
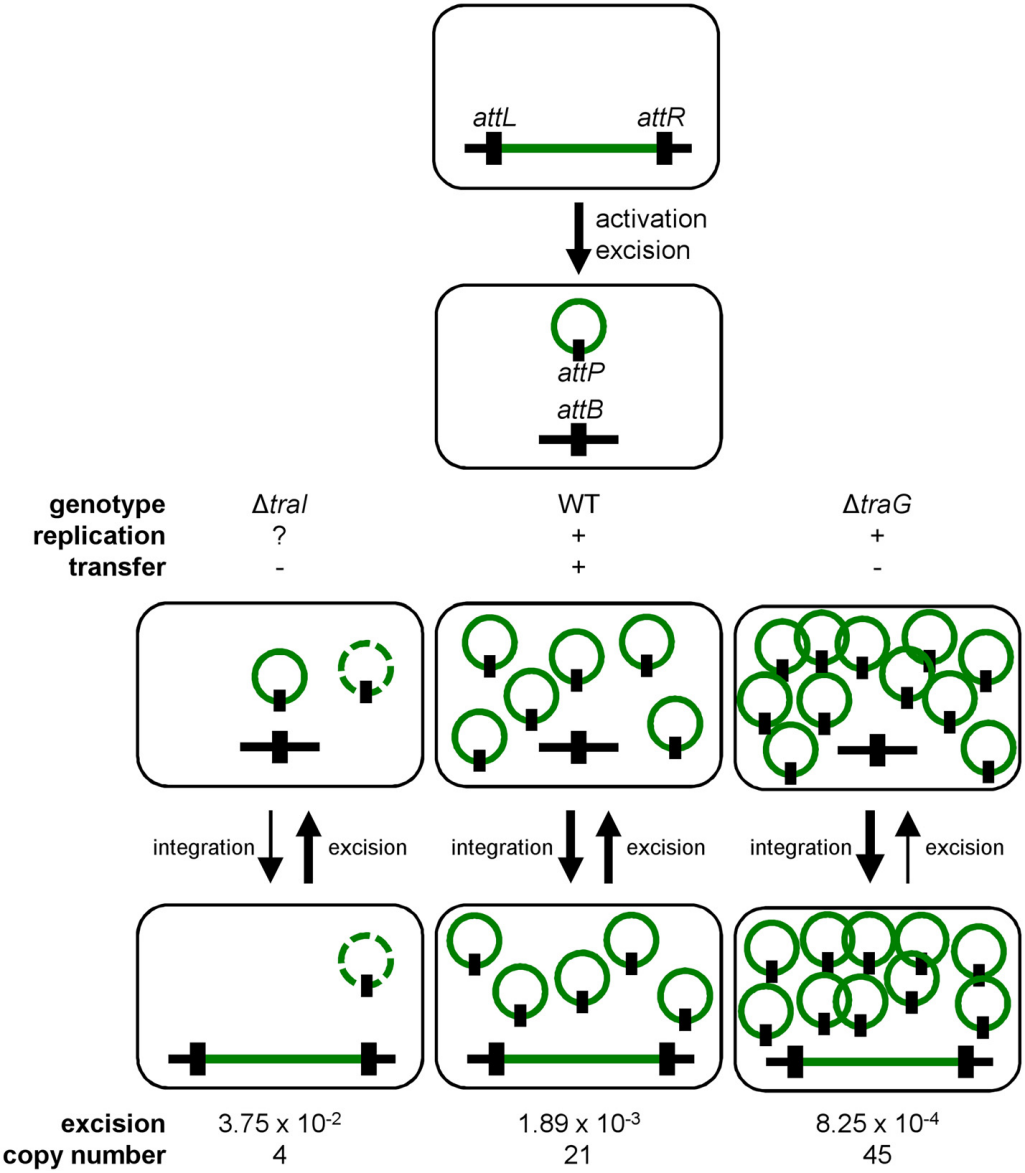
General dynamics of SXT/R391 ICEs. Upon proper stimulation, an integrated ICE is activated and excises from the chromosome. When activated, the ICE is able to replicate and transfer by conjugation. Deletion of *traG* abolishes ICE conjugative transfer due to the non-functional mating pore, and leads to the accumulation of multiple copies resulting from its replication. Deletion of *traI* also abolishes ICE conjugative transfer by inhibiting the initiation of transfer at *oriT* and subsequently impairing rolling-circle replication of the ICE, thereby reducing its copy number. Green line, integrated ICE; green circle, excised ICE, black line, chromosomal integration locus, black rectangle, attachment sites. The dashed green circle represents a putative form of the ICE resulting from low-rate replication by an unknown mechanism.

Conjugation has been shown to be a possible stabilization mechanism for IncP-1 conjugative plasmids in cell populations, allowing the recolonization of plasmid-free cells [36, 62]. However, our results show that conjugation is not a key factor for the stability of SXT/R391 ICEs as a *traG* mutant that is unable to transfer was no less stable than wild-type R391K (Fig 2D). Interestingly, we found that deletion of *traG*, which prevents translocation of the ICE DNA to the recipient cell, had unexpected side effects. In such mutants, the frequency of excision decreased while the extrachromosomal copy number increased (Fig 2A and 2C). A plausible explanation for this phenotype is that plasmid-like molecules of R391K can somehow accumulate due to the blocked mating pore. This accumulation of R391K circles would then tend to displace the site-specific recombination reaction from excision toward reintegration, hence the lower excision frequency (Figs 2A and 7). Consistent with this hypothesis, deletion of *traI* produced the exact opposite effect. We observed that while deletion of *traI* drastically reduced the number of R391K circles, the frequency of excision was very much increased (Fig 3A and 3C). Impaired replication of R391K seems to displace the site-specific recombination reaction equilibrium toward excision instead of integration (Fig 7).

We observed that the frequency of excision does not correlate with the frequency of transfer when comparing SXT and R391. R391 excises at a frequency that is ~10 fold lower than SXT whereas it transfers at a frequency that is ~20 fold higher. We previously reported for SXT that neither its excision from the chromosome of donor cells nor its integration in the chromosome of recipient cells was a step limiting the rate of transfer [14, 26]. This suggests that assembly of the mating apparatus, initiation of transfer or DNA translocation across the cell membranes through the mating pore was the limiting factor. In fact, our data revealed that availability of TraI is a key regulatory element since ovexpression of *traI* in cells bearing R391K Δ*traI* increased the frequency of transfer by 2 logs over wild-type R391K. This observation is supported by RNA-seq data that revealed the relatively low level of expression of *traI* compared to other *tra* genes in SXT, R391 and ICE*Vfl*Ind1, another member of the SXT/R391 family [14]. Therefore, initiation of transfer and/or replication, both depending on TraI and *oriT*, seem to determine the rate of transfer of SXT/R391 ICEs.

Deletion of *traI* or *oriT* drastically reduced the copy number of R391 circles (Fig 3C), which is consistent with a form of replication initiated at *oriT* by the relaxase TraI. The process of conjugation usually relies on an intercellular rolling-circle replication of conjugative elements, making their intracellular replication also virtually possible [8, 63]. Although ICEs were initially defined as non-replicative elements [6], several recent reports strongly support that single-stranded DNA transferring ICEs can replicate as extrachromosomal plasmid-like molecules, in both Gram-positive and Gram-negative bacteria [20–25]. This replication is initiated at *oriT* by the relaxase together with other ICE- and host-encoded auxiliary factors [22, 24]. Notably, the transient replication associated with the conjugative transfer of ICE*Bs1* of *B*. *subtilis*, while not required for transfer, plays an important role in the stability [24]. It relies on *oriT* used as an origin of replication (*oriV*) and on the conjugative relaxase NicK used as the replication initiator protein. Therefore, the rolling-circle replication module being an intrinsic part of the conjugation module, many ICEs, if not all, might be able to transiently replicate as plasmid-like molecules.

Our work revealed yet another intriguing feature of ICEs of the SXT/R391 family besides replication, which seems to blur the frontier between ICEs and plasmids even more. All SXT/R391 ICEs carry *srpMRC*, a locus coding for a functional active partition system. Contrary to low-copy plasmids such as F, which must actively segregate in the daughter cells following cell division, SXT/R391 ICEs usually remain quiescent, integrated into the chromosome of their host, and are passively passed on from one generation to another. Active partition of these ICEs would only be required in their transient excised state, even more so if their copy number per cell is low, such as in the *traI* mutant (Fig 4D). In agreement with this observation, *srpMRC* is part of the same operon coding for the integrase that catalyzes both the integration and excision of SXT/R391 ICEs, all directly under control of the SetCD activator [14]. Therefore, *srpMRC* is expressed only prior to excision, replication and transfer of the ICE. We observed that a Δ*hipA* Δ*traG* Δ*srpM* R391K mutant has an extrachromosomal copy number similar to the wild-type. The apparent suppression of the effect of the Δ*traG* mutation on the extrachromosomal copy number by the loss of *srpM* suggests a link between conjugation and partition that remains to be elucidated.

Active partition of ICEs could be an overlooked feature that is in fact rather common among ICEs. The ICE PAPI-1 of *Pseudomonas aeruginosa* encodes the putative active partition system Soj. Deletion of *soj* leads to high-frequency loss of PAPI-1 [64]. Although the exact mechanism of action of Soj is not well understood, its expression was shown to be stimulated when PAPI-1 excises. ICEs of the pKLC102-ICE*clc* group, including PAPI-1 and ICE*Hin1056*, were shown to be able to replicate and code for putative partitioning systems [21, 23, 65–67]. Moreover, the core region of Tn*4371*-like ICEs and the ICE pNOB8 from *Sulfolobus* codes for ParA and ParB proteins, whose homologs are known to play a role in plasmid partition [68–72]. Finally, ICEA of *Mycoplasma agalactiae* encodes a ParA homolog that could be part of a partitioning system [73]. All these putative partitioning systems could also be involved in incompatibility with other ICEs and/or plasmids, as well as in transcriptional regulation of ICE- and/or host-borne loci [74–76].

Classification of mobile genetic element is extremely laborious mostly because of their modular structure. Our increasingly precise comprehension of their biology unravels some unexpected features that make them even harder to label [77]. On the one hand, ICEs exhibit phage-like behaviors, such as integration by site-specific recombination and, for some ICEs, regulation controlled by CI- or ImmR-like regulators [15, 37, 59, 78, 79]. On the other hand, ICEs also share several characteristics with plasmids, such as a single-strand DNA intermediate during transfer, their conjugative apparatus and entry exclusion systems (*traG*/*eex*) [80, 81]. For instance, the conjugation modules and master activators SetCD and AcaCD of SXT/R391 ICEs and conjugative plasmids of the IncA/C group share a common ancestry [10, 14, 82]. As such SXT/R391 ICEs and IncA/C plasmids offer a dramatic example of divergent evolution from a common ancestor into two different lifestyles. Although SXT/R391 ICEs are capable of transient replication using the relaxase TraI and *oriT*, this lifestyle does not seem to be sustainable over multiple generations [14]. IncA/C plasmids lack the *int* and *xis* genes required for integration and excision, and instead carry a dedicated RepA/C replicon, allowing autonomous, stable and efficient replication in the cell. IncA/C plasmids code for a putative ParMRC-like partitioning system closely related to SrpMRC (*vcrx152*/*vcrx151* in pVCR94) (Fig 6A and 6B). Interestingly, expression of *parMRC*-like locus of SXT/R391 ICEs and IncA/C plasmids is directly under the control of similar yet distantly related class II transcriptional activator complexes: SetCD for SXT/R391 ICEs and AcaCD for IncA/C plasmids [14, 82, 83] (Fig 6B). Given the pleitropic role of these activators, this mode of regulation directly pairs the expression of DNA segregation functions to expression of conjugative transfer functions. However, although IncA/C plasmids retain a type II *parMRC*-like partitioning system (actin-type ATPase), they also carry a type I *parABC*-like partitioning system (Walker-type ATPase) (*vcrx031*/*vcrx032* in pVCR94), which does not seem to be regulated by AcaCD [48, 82, 84, 85]. The exact function and eventual redundancy of each *par* locus remains to be investigated for IncA/C plasmids. The IncHI1 conjugative plasmid R27 also contains two independent partitioning loci, a type I partitioning system, and a type II partitioning system [54]. The type I partitioning system was shown to be the major stability determinant of R27 whereas type II is the minor stability determinant.

Finally, our results put an end to a long standing question: Do SXT/R391 ICEs behave like plasmids and replicate? R391 and related elements such as R705, R748, R997, and pMERPH were initially reported as R factors belonging to the same J incompatibility group (IncJ) [9, 86]. R391 and R997 were even isolated as circular molecules and physically mapped by restriction analysis [19]. Subsequent identification of SXT as an integrative element, and reports of the site-specific integration of R391 and R997 into the same chromosomal site as SXT highlighted seeming incongruities between otherwise extremely similar mobile genetic elements as revealed by sequence comparison [10, 12, 87–90]. In fact, our results indicate that replication, coupled with partition, is a normal yet transitory step of the lifecycle of SXT/R391 ICEs. The transitory nature of this replication does not allow stable maintenance and inheritance as a plasmid-like form. Therefore, integration into the chromosome remains the main mechanism ensuring stable vertical transmission of SXT/R391 ICEs over multiple generations. In the end, despite using similar strategies for their maintenance in the cell population and transfer between cell populations, ICEs and conjugative plasmids remain distinct entities regarding their respective maintenance by integration or replication.

## Materials and Methods

### Bacterial strains and media

The bacterial strains and plasmids used in this study are described in Table 1. The strains were routinely grown in lysogeny broth (LB-Miller, EMD) at 37°C in an orbital shaker/incubator and were preserved at -80°C in LB broth containing 15% (vol/vol) glycerol. Antibiotics were used at the following concentrations: ampicillin (Ap), 100 μg/ml; chloramphenicol (Cm), 20 μg/ml; kanamycin (Kn), 50 μg/ml; mitomycin C (MC), 50 ng/ml; nalidixic acid (Nx), 40 μg/ml; rifampicin (Rf), 50 μg/ml; spectinomycin (Sp), 50 μg/ml; sulfamethoxazole (Su), 160 μg/ml; tetracycline (Tc), 12 μg/ml; trimethoprim (Tm), 32 μg/ml. When required, bacterial cultures were supplemented with 0.02 mM of isopropyl β-D-1-thiogalactopyranoside (IPTG) or 0.02% L-arabinose.

**Table 1:** Strains and plasmids used in this study.

Strains or plasmids	Relevant genotype or phenotype	Source or reference
*E*. *coli*		
HW220	CAG18439 *prfC*::SXT (Tc Su Tm)	[88]
VB111	MG1655 (Nx)	[38]
VB112	MG1655 (Rf)	[38]
CAG18439	MG1655 *lacZU118 lacI42*::Tn*10* (Tc)	
VB38	CAG18439 Δ*galK* (Tc)	[18]
VB71	CAG18439 Δ*lacZ* (Tc)	[18]
TOP10F'	F'[*lacI*q Tn*10*(Tc)] *mcrA* Δ(*mrr-hsdRMS-mcrBC*) Φ80*lacZ*Δ*M15* Δ*lacX74 deoR nupG recA1 araD139* Δ(*ara-leu*)*7697 galU galK rpsL*(Sm) *endA1* λ-	Invitrogen
VI61	CAG18439 Δ*lacZ*::*attP-cat*	[26]
GG13	VB38 R391K (Tc Kn)	[18]
NC174	VB38 R391K[Δ*hipA*::*aad7*] (Tc Kn Sp)	This study
NC187	VB38 R391K[Δ*traG*::*cat*] (Tc Kn Cm)	This study
NC195	VB38 R391K[Δ*hipA*::*aad7*] Δ*traG*::*cat* (Tc Kn Sp Cm)	This study
NC186	VB38 R391K[Δ*traI*::*cat*] (Tc Kn Cm)	This study
NC190	VB38 R391K[Δ*hipA*::*aad7* Δ*traI*::*cat*] (Tc Kn Sp Cm)	This study
NC175	VB38 R391K[Δ*hipA*::*aad7* Δ*srpR*] (Tc Kn Sp)	This study
NC176	VB38 R391K[Δ*hipA*::*aad7* Δ*srpM*] (Tc Kn Sp)	This study
NC177	VB38 R391K[Δ*hipA*::*aad7* Δ*srpRM*] (Tc Kn Sp)	This study
NC197	VB38 R391K[Δ*hipA*::*aad7* Δ*traG*::*cat* Δ*srpM*] (Tc Kn Sp Cm)	This study
NC192	VB38 R391K[Δ*hipA*::*aad7* Δ*traI*::*cat* Δ*srpM*] (Tc Kn Sp Cm)	This study
NC92	VB71 R391 Δ*srpR* (Kn)	This study
NC93	VB71 R391 Δ*srpM* (Kn)	This study
NC94	VB71 R391 Δ*srpRM* (Kn)	This study
DC60	VB111 R391 Δ*oriT* (Nx Kn)	[38]
Plasmids		
pSIM5	Thermo-inducible expression of λRed recombination (Ts Cm)	[91]
pSIM6	Thermo-inducible expression of λRed recombination (Ts Ap)	[91]
pKD3	Cm template for one-step chromosomal gene inactivation	[92]
pKD4	Kn template for one-step chromosomal gene inactivation	[92]
pVI36	Sp template for one-step chromosomal gene inactivation	[38]
pAH56	*oriV*R6Kγ; *attP*λ; *lacI*; *P*tac-*uidAF* (Kn)	[93]
pVB15	*oriV*pMB1; *lacI*; *P*tac-*uidAF* (Kn)	This study
ps002-his	pVB15 Δ*uidAF*::*srpR*SXT (Kn)	This study
pTraI	pBAD-TOPO::*traI*SXT (Ap)	This study
pTraG	pBAD-TOPO::*traG*R391 (Ap)	This study
pBeloBAC11	single-copy vector derived from the F plasmid (Cm)	New England Biolabs
pBeloBAC11Δ*sop*	pBeloBAC11 Δ*sopABC* (Cm)	This study
pSrpSXT	pBeloBAC11Δ*sop*::*srpMRC*SXT (Cm)	This study
pSrpR391	pBeloBAC11Δ*sop*::*srpMRC*R391 (Cm)	This study
pSrpR391ΔR	pBeloBAC11Δ*sop*::*srpMC*R391 (Cm)	This study
pSrpR391ΔM	pBeloBAC11Δ*sop*::*srpRC*R391 (Cm)	This study
pSrpR391ΔC	pBeloBAC11Δ*sop*::*srpMR*R391 (Cm)	This study

Ap, ampicillin; Cm, chloramphenicol; Kn, kanamycin; Sp, spectinomycin; Sm, streptomycin; Su, sulfamehoxazole, Tc, tetracycline; Tm, trimethoprim, Ts, thermosensitive.

### Bacterial conjugation assays

Conjugation assays were performed by mixing equal volumes of each donor and recipient strains that were grown overnight at 37°C. The cells were harvested by centrifugation for 3 min at 1200g, washed in 1 volume of LB broth and resuspended in 1/20 volume of LB broth. Mating mixtures were then deposited on LB agar plates and incubated at 37°C for 6 hours. The cells were recovered from the plates in 1 ml of LB broth and serially diluted before plating. Donors, recipients and exconjugants were selected on LB agar plates containing appropriate antibiotics.

### Molecular biology methods

Plasmid DNA was prepared using the EZ-10 Spin Column Plasmid DNA Minipreps Kit (Biobasic) according to manufacturer’s instructions. All the enzymes used in this study were purchased from New England BioLabs. PCR assays were performed with the primers described in S1 Table. The PCR conditions were as follows: (i) 3 min at 94°C; (ii) 30 cycles of 30 sec at 94°C, 30 sec at the appropriate annealing temperature, and 1 minute/kb at 68°C; and (iii) 5 min at 68°C. When necessary, PCR products were purified using an EZ-10 Spin Column PCR Products Purification Kit (Biobasic) according to manufacturer’s instructions. *E*. *coli* was transformed by electroporation as described by Dower *et al*. [94] in a BioRad GenePulser Xcell apparatus set at 25 μF, 200 V and 1.8 kV using 1-mm gap electroporation cuvettes. Sequencing reactions were performed by the Plateforme de Séquençage et de Génotypage du Centre de Recherche du CHUL (Québec, QC, Canada).

### Plasmid and strain construction

Plasmids and oligonucleotides used in this study are listed in Table 1 and S1 Table. pTraI and pTraG were constructed by cloning *traI* of SXT and *traG* of R391 into the TA cloning expression vector pBAD-TOPO (Invitrogen) according to the manufacturer’s instructions. *traI* was amplified by PCR with its native Shine-Dalgarno sequence using primers pBad-traI_Fw and pBad-traI_Rev and genomic DNA of *E*. *coli* HW220 as the template. *traG* was amplified using the primer pair traGEcoRI.for / traGEcoRI.for and genomic DNA of *E*. *coli* GG13 as the template. pVB15 was constructed by amplifying the origin of replication of pUC19 (*oriV*_pMB1_) using the primer pair pUC_oriF/pUC_oriR and subsequent cloning into the 5 838-bp fragment of NheI/NotI-digested pAH56 to replace *oriV*_R6K_-*attP*_λ_ and generate the high-copy number expression vector pVB15. p*s002*-his was then obtained by cloning *s002* (*srpR*) from SXT amplified with the primer pair s002F/s002-hisR into the 4 319-bp fragment of NdeI/BamHI-digested pVB15.

Plasmids used for plasmid stabilization assays were derived from pBeloBAC11Δ*sop*, a pBeloBAC11 vector derivative from which the partitioning system *sopABC* was deleted by NdeI digestion and re-ligation. The *srpMRC* locus of SXT (*srpMRC*_SXT_) and R391 (*srpMRC*_R391_) were amplified by PCR using genomic DNA of strains containing either SXT or R391 as the templates and primers pairs SXTpartHindIIIstop.for/SXTR391partHindIII.rev and R391partHindIIIstop.for/SXTR391partHindIII.rev, respectively. Amplicons were digested by HindIII and cloned into HindIII-digested pBeloBAC11Δ*sop* to generate pSrp_SXT_ and pSrp_R391_. Subsequent deletions of segments of *srpMRC*_R391_ were obtained by high fidelity PCR amplification of the pSrp_R391_ vector using primer pairs pBeloDelSO02.for/pBeloDelSO02.rev, pBeloDelSO03.for/pBeloDelSO03.rev or pBeloDelparC.for/pBeloDelparC.rev, digestion by NheI and ligation using the T4 DNA ligase. The resulting plasmids were verified by restriction profiling and DNA sequencing.

Deletion mutants of R391::*galK* (R391K) [18] were constructed using the one-step chromosomal gene inactivation [92] and P1*vir* transduction [95] techniques. Deletion of *hipA*, *srpR*, *srpM*, *srpRM*, *traI* and *traG* were constructed using primer pairs R391DhipAnoFRT.for/R391DhipAnoFRT.rev, 2SXTR391DSO02.for/2SXTR391DSO02.rev, R391DSO03.for/2SXTR391DSO03.rev, R391DSO03.for/2SXTR391DSO02.rev, R391DtraInoFRT.for/R391DtraInoFRT.rev, R391DtraGnoFRT.for/R391DtraGnoFRT.rev, respectively. Gene resistance cassettes were amplified using the pVI36, pKD3 and pKD4 vectors. The λRed recombination system was expressed using pSIM5 or pSIM6 as described by Datta *et al*. [91]. If possible, the antibiotic resistance cassette was removed from the resulting construction by Flp-catalyzed excision using the pCP20 vector [96]. All deletions were verified by PCR and antibiotic resistance profiling.

### ICE and plasmid stability assays

The stability of R391::*galK* and derivative mutants was monitored based on the methodology described by Wozniak and Waldor [32]. Cells were grown for 16 hours in 4 ml of LB medium supplemented or not with kanamycin. Serial dilutions were plated on MacConkey agar plates supplemented with 1% D-galactose. Loss of R391 resulted in the formation of white clones (Fig 1C). For each experiment, at least 16 white clones were purified and tested on agar plate for their susceptibility to kanamycin. These clones were also tested by PCR amplification of an internal fragment of R391 using primer pair R391HipBM1.for/R391HipB.rev. The stability of pBeloBAC11Δ*sop* and derivatives containing the *srpMRC* locus of SXT or R391 was tested for 16 hours in M9 or LB medium using the approach described by Sanchez *et al*. [97]. Expression of the *srp* locus from *P*_*lac*_ was induced by addition of 0.02 mM IPTG. Relative stability was calculated as the ratio of chloramphenicol resistant colonies in the population in the induced compared to the non-induced conditions. For both ICE and plasmid stability assays, each experiment was carried out at least in biological triplicate.

### Determination of ICE dynamics using real-time quantitative PCR

The frequency of excision as well as total copy number in the population and copy number of the excised circular form of the ICE were assessed by real-time quantitative PCR as described elsewhere [20, 26]. Genomic DNA was obtained from cell cultures of *E*. *coli* CAG18439 bearing SXT, R391K or its mutants grown for 16 h in LB medium. *prfC*, *attB*, *attP* and *int* were quantified using primer pairs prfC.qec.F1/prfC.qec.R1, attB.qec.F2/attB.qec.R2, attP.qec.F2/attP.qec.R2 and int.qec.F1/int.qec.R1, respectively (S1 Table). For frequency of excision and copy number determination, *E*. *coli* VI61, which contains one chromosomal copy of *attB*, *attP* and *prfC*, was used to simulate 100% of excision and normalize the results. qPCR experiments were performed in triplicate on the RNomics platform of the Laboratoire de Geénomique Fonctionnelle de l’Universiteé de Sherbrooke (http://lgfus.ca) (Sherbrooke, QC, Canada).

### Macroscopic observations of colonies

Macroscopic observations were done using a SZX7 zoom stereomicroscope with a DF PLAPO1X-4 objective coupled to a SC30 digital camera *via* a U-TV1X-2 & U-CMAD3 adaptor (Olympus).

### SrpR expression and purification

To express and purify SrpR tagged with a 6×His C-terminal epitope (SrpR^6×His^), cultures of *E*. *coli* BL21 bearing ps002-his were grown overnight, diluted 1:500 in fresh 2×YTA broth and incubated at 37°C with agitation. At mid-exponential phase (OD_600_ of 0.6), protein expression was induced with 0.1 mM IPTG and cultures were incubated for 3 hours. Cells were then harvested by centrifugation at 1500×g for 10 min at 4°C and stored at -20°C. The cell pellet was weighted and re-suspended in Native Purification Buffer (NPB) (50 mM NaH_2_PO_4_ pH 8.0, 2.5 M NaCl) containing 0.1% Triton X-100, 1 mM phenylmethanesulfonylfluoride (PMSF), and protease inhibitors at 1 ml / 20g of cell pellet (Protease Inhibitor Cocktail, Sigma). Purification of SrpR^6×His^ was done by Ni-NTA affinity chromatography following the manufacturer’s instructions (Qiagen). Cells were lysed by sonication, cell debris was pelleted by centrifugation, and the supernatant was incubated for 1 h at 4°C with 750 μl of Ni-NTA Agarose resin (QIAGEN) with agitation. The Ni-NTA Agarose resin was then transferred into a column and washed 4 times with 1.25 ml of native wash buffer (NPB with 20 mM imidazole, pH 8.0). SrpR^6×His^ was eluted with native elution buffer (NPB with 250 mM imidazole, pH 8.0) and stored at -20°C. Protein concentration was estimated using a Bradford protein assay (BioRad) and purity was determined by SDS-PAGE analysis.

### Electrophoretic mobility shift assays (EMSA)

The linear double-stranded DNA probes *srpC* (251 bp), *oriT* (298 bp) and *ig(srpM-mobI)* (615 bp) used in the EMSA experiments were amplified by PCR using primer pairs RRintF/RRintR, oriT2F/oriT2R and MELR1/oriT1R, respectively, and *E*. *coli* HW220 as the template (Table 1 and S1 Table). Probes were purified using an EZ-10 Spin Column PCR Products Purification Kit (Bio Basic) according to the manufacturer's instructions and their concentration was determined using a NanoDrop ND-1000. Probes R3-R1, R4-R2, R2 and R4 were obtained by mixing equimolar concentrations (50 μM) of primers, srpCSXTR3R1F and srpCSXTR3R1R, srpCSXTR4R2F and srpCSXTR4R2R, srpCSXTR2F and srpCSXTR2R, or srpCSXTR4F and srpCSXTR4R, respectively. The primer mixtures were heated at 95°C for 3 min, then annealed by slow cool down overnight.

EMSA assays were carried out using the Electrophoretic Mobility-Shift Assay Kit with SYBR Green & SYPRO Ruby EMSA stains (Life Technologies) according to the manufacturer's instructions. Briefly, a total of 40 ng of DNA probe was used in each reaction. Quantities of SrpR^6×His^ and of the non-specific competitor DNA (sonicated salmon sperm DNA) varied from 10 to 100 ng, and 10 to 200 pg, respectively. The non-specific competitor DNA was mixed with the probe before adding SrpR^6×His^ to maximize competition. All binding reactions were done in a total volume of 10 μl for 15 min at room temperature followed by 10 min incubation on ice. Samples were then loaded on a pre-run (25 min at 100 V) non-denaturing 4% acrylamide gel containing 1× TBE buffer and migration was carried out at 4°C during electrophoresis. SYBR Green staining was done according to the manufacturer's instructions and gel pictures were scanned using a Typhoon FLA 9500 (GE Healthcare Life Sciences) with a LPB filter for SYBR Green I at a 100 μm resolution.

### Dimerization assay

Dimerization assays were carried out using 2 μg of purified SrpR^6×His^. Samples containing *srpC* were carried out using 1 μg of DNA probe and were incubated prior to the dimerization assay in the same conditions as for the EMSA assays. Samples were incubated with or without 0.6% glutaraldehyde for 30 min at room temperature and 3% of *β*-mercaptoethanol was added to the samples prior to denaturation at 95°C for 3 min. Samples and ladder (Precision Plus Protein Kaleidoscope Standards, BioRad) were separated by electrophoresis on a 12% SDS-PAGE gel, later stained using Coomassie Brilliant Blue R-250.

### Phylogenetic analyses

The molecular phylogenetic analysis of SrpM was conducted in MEGA6 [98] The primary sequence of SrpM encoded by R391 was used to search for homologous sequences in the Genbank Non-redundant protein sequence (nr) database using blastP [99]. Phylogenetic analyses were computed using a protein alignment generated by MUSCLE [100] and poorly aligned regions were removed with the trimAl v1.3 software using the automated heuristic approach [101] prior to phylogenetic analyses. The evolutionary history was inferred by using the Maximum Likelihood method. Initial tree(s) for the heuristic search were obtained automatically by applying Neighbor-Join and BioNJ algorithms to a matrix of pairwise distances estimated using a JTT model, and then selecting the topology with superior log likelihood value. A discrete Gamma distribution was used to model evolutionary rate differences among sites (5 categories (+*G*, parameter = 4.1321)). The rate variation model allowed for some sites to be evolutionarily invariable ([+*I*], 2.6007% sites). The tree is drawn to scale in iTOL v2 [102], with branch lengths measured by the number of substitutions per site. The analysis involved 60 amino acid sequences with a total of 261 positions in the final dataset.

## Supporting Information

S1 Fig Plasmid stabilization assays in fast growing conditions. The experimental conditions of the assay were similar to those described in Fig 4E, except that the cells were grown in LB medium.
(TIF)

S1 Table Primers used in this study. (DOCX)
