# Supplementary material for: Replication and Active Partition of Integrative and Conjugative Elements (ICEs) of the SXT/R391 Family: The Line between ICEs and Conjugative Plasmids Is Getting Thinner
Source: PLoS Genet. 2015 Jun 10;11(6):e1005298. doi: 10.1371/journal.pgen.1005298 (PMC4489591; doi:10.1371/journal.pgen.1005298)
Supplement: S1 Table — (DOCX) [file pgen.1005298.s002.docx]

## Table S1: Primers used in this study

| **Name** | **Nucleotide sequence (5’ to 3’)*^a^*** |
| --- | --- |
| prfC.qec.F1 | AGTCAACGTTGCCACTGCCC |
| prfC.qec.R1 | AAGCGCCAGTTGGCTTTCGT |
| attB.qec.F2 | GCGCGATGCCGCTTACTCAA |
| attB.qec.R2 | GCGGTCTGAATGGCCTGTCC |
| attP.qec.F2 | AGATCAGCGAAAATAGCGGCCA |
| attP.qec.R2 | GCGTTGAAAGGCTGGGCGG |
| int.qec.F1 | GCCGATCATGCGGCCATTGA |
| int.qec.R1 | ACACCAAGCGATCTGCGAGC |
| 2SXTR391DSO02.for | TGCTTCTTTATTTAACCTGGCATCGAGCCAGCTAACTGATAACGCCATATGAATATCCTCCTTA |
| 2SXTDR391SO02.rev | TGTTTTGGCGAACGTCCGAGGCTTCTGGTTTTATGGGGCCTGATTGTGTAGGCTGGAGCTGCTTC |
| R391DSO03.for | GAGTAGTTCAGGGTGGCTCCCTGGGGGAATGTTGACGACAACTCTCATATGAATATCCTCCTTA |
| 2SXTR391DSO03.rev | TTGATTGAAAACCGTTAGAGCACTTTGAGTGCACAGGAGATAAGTGTGTAGGCTGGAGCTGCTTC |
| R391DhipAnoFRT.for | GCCCGCGTATCAGTTAGAGGAAGACGACGATGACTAACTAATAAAGAGCGCTTTTGAAGCTCA |
| R391DhipAnoFRT.rev | ACCATCTTTATCGATATGCACGCTCTAGCTACTAGGTATCGGAATAGGAACTTCAAGAAT |
| R391DtraInoFRT.for | CAGAGTTGAACCTTCACACTATCGTGTCGGAGGTTCACTCGCGCCTACCTGTGACGGAA |
| R391DtraInoFRT.rev | GCAAAATAACCGTTTGCAATCCACTTAAGTTCAAATGGAGGAATAGGAACTTCATTTAA |
| R391DTraGnoFRT.for | TATTACACCGTTAAATCTACGGCACTGGGGGAATAGGTCGGAATAGGAACTTCATTTAA |
| R391DTraGnoFRT.rev | AGCCATACACGAGCATGAGCGAAATTGCCGGAACACTCGCGCGCCTACCTGTGACGGAA |
| SXTpartHindIIIstop.for | NNNNNNAAGCTTTAAATAAAATAAGTCATCCGCCGCCAAACAGA |
| R391partHindIIIstop.for | NNNNNNAAGCTTTAAATAAAATAAGTCATCCACCGCCAAACAGA |
| SXTR391partHindIII.rev | NNNNNNAAGCTTTTATAAGCCCAGCCCTAGTTT |
| pBeloDelSO02.for | NNNNNNGCTAGCTCAGGCCCCATAAAACCAGA |
| pBeloDelSO02.rev | NNNNNNGCTAGCGCCTGCAGGTCGACTCTA |
| pBeloDelSO03.for | NNNNNNGCTAGCACTTATCTCCTGTGCACTCAA |
| pBeloDelSO03.rev | NNNNNNGCTAGCATGAGAGTTGTCGTCAACATT |
| pBeloDelparC.for | NNNNNNGCTAGCTTATTTTATTTAAAGCTTGAGTATTCTA |
| pBeloDelparC.rev | NNNNNNGCTAGCGTGCACAGGAGATAAGTATGTT |
| pBad-traI_Fw | TAGTGTCGGAGGTTCACTATGTTCAAAAACCTAT |
| pBad-traI_Rev | TTAACGTTTAGCGTGAGCGGTAAAC |
| traGEcoRI.for | TAATAAGAATTCAAGGAGGAATAATAAATGTGGGAGATCTATTCCAT |
| traGEcoRI.rev | NNNNNNGAATTCTTATTTATTTCCTGATGCCTGGTT |
| pUC_oriF | NNNNNNGCGGCCGCGTTGCTGGCGTTTTTCCA |
| pUC_oriR | NNNNNNGCTAGCGTCAGACCCCGTAGAAAAGA |
| s002F | NNNNNNCATATGAGAGTTGTCGTCAACATT |
| s002-hisR | NNNNNNGGATCCTTAGTGGTGATGGTGATGATGAGAACCACGTAAGCCCAGCCCTAGTTTGGATTTTAA |
| MELR1  oriT2F  oriT1R  RRintF | CGCTCTAGAATACGATCCGCAGGA  GCTCTAGATGGCGGCGGATGA  TTTCTAGAAAACCAATTTCCCCA  ACATACTTATCTCCTGTGCACTCA |
| RRintR | ACGACGTTTGGCGTCTCGAT |
| srpCSXTR4R2F | TACTTATCTCCTGTGCACTCAAAGTGCTCTAACGGTTTTC |
| srpCSXTR4R2R | GAAAACCGTTAGAGCACTTTGAGTGCACAGGAGATAAGTA |
| srpCSXTR4F | ACAAACATACTTATCTCCTGTGCACTCAGTTTTCAATCAA |
| srpCSXTR4R | TTGATTGAAAACTGAGTGCACAGGAGATAAGTATGTTTGT |
| srpCSXTR2F | CAAACATACTTATAAAGTGCTCTAACGGTTTTCAATCAAC |
| srpCSXTR2R | GTTGATTGAAAACCGTTAGAGCACTTTATAAGTATGTTTG |
| srpCSXTR3R1F | TGGCCACGCTTGGGCATTTCCAAGTGCTCTGGCGGTTCAC |
| srpCSXTR3R1R | GTGAACCGCCAGAGCACTTGGAAATGCCCAAGCGTGGCCA |

*^a^* Restriction sites are underlined
